# Ternary metal fluorides as high-energy cathodes with low cycling hysteresis

**DOI:** 10.1038/ncomms7668

**Published:** 2015-03-26

**Authors:** Feng Wang, Sung-Wook Kim, Dong-Hwa Seo, Kisuk Kang, Liping Wang, Dong Su, John J. Vajo, John Wang, Jason Graetz

**Affiliations:** 1 Sustainable Energy Technologies Department, Brookhaven National Laboratory, Upton, New York 11973, USA; 2 Department of Materials Science and Engineering, Research Institute of Advanced Materials, Seoul National University, Seoul 151-742, Republic of Korea; 3 Center for Nanoparticle Research, Institute for Basic Science, Seoul National University, Seoul 151-742, Republic of Korea; 4 Center for Functional Nanomaterials, Brookhaven National Laboratory, Upton, New York 11973, USA; 5 Sensors and Materials Laboratory, HRL Laboratories, LLC, Malibu, California 90265, USA

## Abstract

Transition metal fluorides are an appealing alternative to conventional intercalation compounds for use as cathodes in next-generation lithium batteries due to their extremely high capacity (3–4 times greater than the current state-of-the-art). However, issues related to reversibility, energy efficiency and kinetics prevent their practical application. Here we report on the synthesis, structural and electrochemical properties of ternary metal fluorides (M^1^_*y*_M^2^_1-*y*_F_*x*_: M^1^, M^2^=Fe, Cu), which may overcome these issues. By substituting Cu into the Fe lattice, forming the solid–solution Cu_*y*_Fe_1-*y*_F_2_, reversible Cu and Fe redox reactions are achieved with surprisingly small hysteresis (<150 mV). This finding indicates that cation substitution may provide a new avenue for tailoring key electrochemical properties of conversion electrodes. Although the reversible capacity of Cu conversion fades rapidly, likely due to Cu^+^ dissolution, the low hysteresis and high energy suggest that a Cu-based fluoride cathode remains an intriguing candidate for rechargeable lithium batteries.

## Abstract (web summary)

Transition metal fluorides have high theoretical specific capacities as cathodes for lithium ion batteries, but low working potentials and poor energy efficiency limit their practical applications. Here, the authors report a group of ternary metal fluorides, which may overcome these problems.

## Graphical abstract

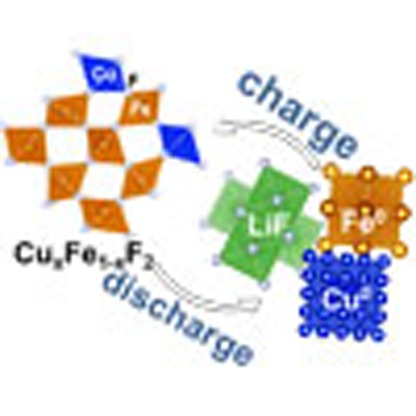

Lithium ion batteries (LIBs) are the preferred energy storage devices for portable electronics, and their use in electric vehicles and grid-level energy storage is increasing rapidly^1^,^2^,^3^. However, large-scale application requires greater energy density per unit cost (by two times or more) for widespread use. The capacity of conventional cathodes (for example, LiCoO_2_, LiFePO_4_) is low (140–170 mAh g^−1^) and currently limits the energy density of most commercial cells. Although a number of alternative anodes (such as Si and Sn) exhibit capacities well above 500 mAh g^−1^, few cathodes have been identified that can match such high capacity. The conversion cathodes, specifically the fluoride-based materials, are an exception to this rule and exhibit extremely high specific capacities, enabled by more than one electron transfer per transition metal (M^n+^X_y_+nLi^+^+ne^−^=yLi_n/y_X+M^0^; *n*≥*2*)^4^,^5^,^6^,^7^, in addition to their intrinsically high redox potentials (>2 V)^5^,^8^,^9^,^10^,^11^,^12^,^13^,^14^,^15^,^16^,^17^,^18^. CuF_2_ is particularly attractive because of its extremely high theoretical potential (~3.55 V) and specific capacity (~528 mAh g^−1^), offering an exceptionally high specific energy density (1,874 Wh kg^−1^)^8^,^9^. However, the electrochemical activity of CuF_2_ is low, and utilization of its full capacity was only recently achieved by embedding CuF_2_ into a conductive matrix^13^. Unfortunately, the utility of CuF_2_ has been limited to primary batteries due to the irreversibility of the Cu^2+/0^ redox reaction. Other fluorides, such as FeF_2_ and FeF_3_ exhibit high reversibility^15^,^16^,^17^,^18^, but their low working potentials and poor energy efficiency (due to large polarization and cycling hysteresis), continue to limit their practical use in commercial batteries.

Recently, extensive research on metal fluoride cathodes has provided new insights into the mechanisms involved in the conversion reactions and the issues relevant to cycling reversibility and efficiency (for example, hysteresis)^8^,^9^,^10^,^11^,^12^,^13^,^14^,^15^,^16^,^17^,^18^,^19^,^20^,^21^. Although poor electronic and ionic transport plague many conversion electrodes, recent studies show that the electronic conductivity in FeF_2_ improves lithiation and approaches that of metallic Fe (ref. 20). The percolating Fe network formed during lithiation provides a facile electronic pathway^15^,^16^,^19^,^20^, and the high interfacial area provides abundant pathways for rapid Li^+^ transport^15^,^22^. In contrast, the conversion reaction in CuF_2_ involves highly mobile Cu^2+^ ions, which leads to coarsening and growth of large, isolated Cu particles during lithiation, making reconversion difficult^15^,^17^. In addition, a recent study of the CuF_2_ conversion reaction by Hua *et al*.^23^, clearly showed that the dominant reaction occurring during the 1st charge is the dissolution of Cu into the electrolyte to form an unidentified Cu^+^ species, resulting in considerable loss of capacity. An intriguing new concept, derived from these recent findings, is the possibility of substituting Cu into the Fe fluoride system, and thereby forming a ternary solid–solution Cu_*y*_Fe_1-*y*_F_2_. An electrode configured in this way would potentially benefit from the percolating iron network, which may be effective at ‘trapping’ Cu ions allowing them to fully oxidize into Cu^2+^. The addition of a second cation into a solid–solution is also an effective strategy for tailoring electrochemical properties (thermodynamics and kinetics) and improving electrochemical performance, as already demonstrated in many electrodes^24^,^25^,^26^,^27^,^28^. Surprisingly, despite tremendous research on the binary metal fluorides^8^,^9^,^10^,^11^,^12^,^13^,^14^,^15^,^16^,^17^,^18^,^19^,^20^, studies of conversion reactions in the ternary fluorides (involving two transition metal cations) have been largely overlooked.

In this study, solid solutions of the ternary metal fluorides M^1^_*y*_M^2^_1-*y*_F_*x*_ (M^1^, M^2^=transition metal), were prepared via mechanochemical reactions. The structure, stability and electrochemical properties of Cu_*y*_Fe_1-*y*_F_2_ were investigated by density functional theory (DFT) calculations, electrochemical measurements, along with comprehensive structural and chemical analysis using synchrotron X-ray diffraction (XRD), absorption spectroscopy (XAS) and (scanning) transmission electron microscopy ((S)TEM) coupled with electron energy loss spectroscopy (EELS). Electrochemical measurements indicated a reversible Cu redox reaction (that is, Cu^2+/0^) in the mixed system, Cu_0.5_Fe_0.5_F_2_, in contrast to irreversible behaviour observed in the binary fluoride, CuF_2_ (ref. 23). This result was subsequently confirmed by XAS and TEM–EELS measurements. The voltage hysteresis for the Cu redox (Cu^2+/0^) in Cu_*y*_Fe_1-*y*_F_2_ is surprisingly small, <148 mV, which is likely to be the lowest value ever measured for conversion reactions in metal fluorides. A comprehensive investigation of the reaction mechanisms, thermodynamics and kinetics of the lithium (re)conversion reactions in the solid–solution Cu_*y*_Fe_1-*y*_F_2_ reveals that the incorporation of Cu into the Fe lattice enables a cooperative redox reaction, which leads to the reversible Cu redox (Cu^2+^←Cu^0^).

## Results

### Structure of ternary metal fluorides

**Figure 1:**
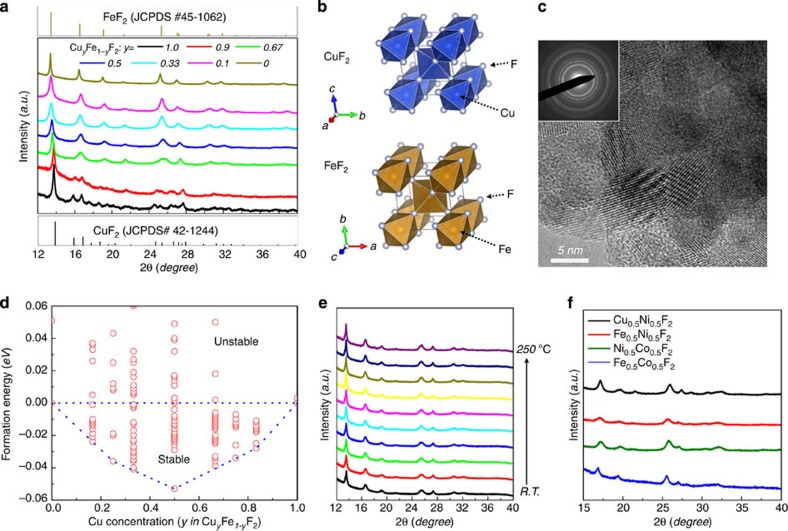
Structure and stability of novel ternary metal fluorides. (**a**) Synchrotron XRD patterns from Cu_*y*_Fe_1-*y*_F_2_ along with CuF_2_ (JCPDS#42–1244) and FeF_2_ (JCPDS#45–1062), (**b**) schematic illustration of the FeF_2_ (rutile) and CuF_2_ (distorted rutile) structures, (**c**) high-resolution TEM image of as-synthesized Cu_0.5_Fe_0.5_F_2_ nanocrystallites (inset: electron diffraction pattern from a large area), (**d**) energy diagram of Cu_*y*_Fe_1-*y*_F_2_ phases (at various possible configurations) predicted by DFT calculations, (**e**) *in situ* XRD patterns recorded during heating of Cu_0.5_Fe_0.5_F_2_ from room temperature (R.T.) to 250 °C, (**f**) XRD patterns from representative ternary fluorides of varying metal species, M^1^_0.5_M^2^_0.5_F_2_ (M^1^, M^2^=Cu, Fe, Ni, Co).

The crystal structures of as-synthesized M^1^_*y*_M^2^_1-*y*_F_2_ powders were examined using synchrotron XRD and TEM. Figure 1a shows the XRD patterns of the Cu_*y*_Fe_1-*y*_F_2_ system at several different Cu/Fe ratios (*y*=0, 0.1, 0.33, 0.5, 0.67, 0.9, 1). The broadened diffraction peaks indicate a loss of long-range order during the mechanochemical synthesis. Interestingly, the milling of CuF_2_ and FeF_2_ precursors leads to the formation of a single solid–solution phase over the entire compositional range. This is not too surprising given the similarity of the CuF_2_ and FeF_2_ structures. FeF_2_ exhibits a tetragonal rutile structure (space group: P4_2_/*mnm*) and is comprised of FeF_6_ octahedra, while CuF_2_ is monoclinic (space group: P2_1_/*n*), which is essentially a distorted rutile structure due to the strong Jahn–Teller distortion induced by the Cu^2+^ ([Ar]3d^9^) ion (Fig. 1b and Supplementary Fig. 1)^27^. The distorted structure of CuF_2_ becomes more symmetric with Fe incorporation as the Cu_*y*_Fe_1-*y*_F_2_ solid–solution is formed (see Supplementary Fig. 2 and Supplementary Note 1). The as-synthesized samples are complex agglomerates of small nanocrystallites as shown by brightfield TEM image (<10 nm; Fig. 1c). The diffusive rings in the electron diffraction pattern (although being broadened due to the nanocrystalline nature of the particles; inset in Fig. 1c) can be assigned to the tetragonal rutile phase (Supplementary Fig. 3), consistent with the XRD measurements (Fig. 1a).

DFT calculations were used to predict the stability of solid–solution phases, at all the possible configurations (see details in Methods below). The energy difference between the possible Cu_*y*_Fe_1-*y*_F_2_ phases and the simple *y*CuF_2_-(1*-y)*FeF_2_ mixture (Fig. 1d) indicates that, regardless of the composition, there exist several Cu_*y*_Fe_1-*y*_F_2_ phases that are energetically more stable (negative energy points) than the simple mixture (zero energy points). The lowest energy points at each composition overlap well with the convex hull (dashed line), indicating that Cu_*y*_Fe_1-*y*_F_2_ can exhibit solid–solution behaviour over the entire composition range. The structural stability of the solid–solution phase was experimentally confirmed by *in situ* XRD (Fig. 1e), which shows no phase decomposition in Cu_0.5_Fe_0.5_F_2_ during dynamic heating up to 250 °C.

Since most of the 3d metal binary fluorides (that is, MF_2_) have similar structures, either based on the tetragonal rutile or distorted rutile framework, it is expected that they may form a variety of solid solutions. A number of ternary fluoride phases were prepared, including Cu_0.5_Ni_0.5_F_2_, Fe_0.5_Ni_0.5_F_2_, Ni_0.5_Co_0.5_F_2_ and Fe_0.5_Co_0.5_F_2_ (Fig. 1f), which demonstrates the versatility of the mechanochemical synthesis method.

### Electrochemical properties of Cu_*y*_Fe_1-*y*_F_2_

**Figure 2:**
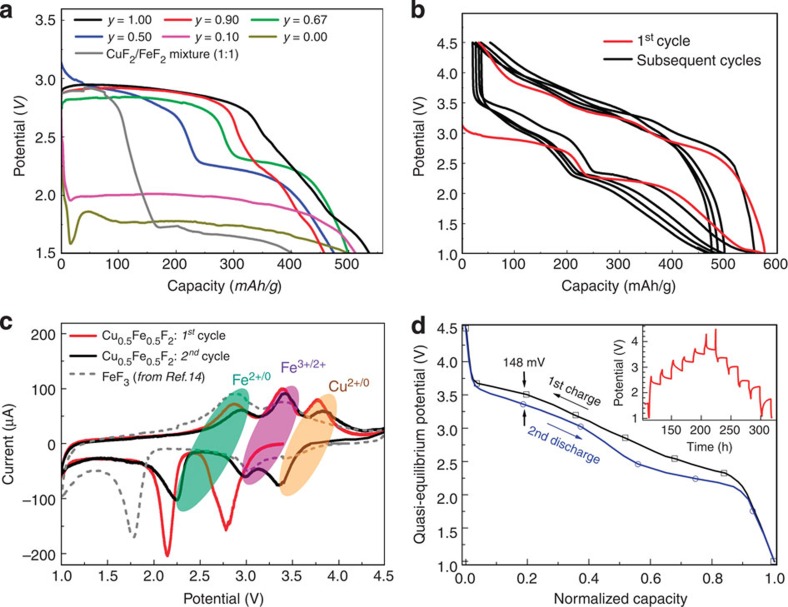
Electrochemical properties of Cu_*y*_Fe_1-*y*_F_2_ solid–solution. (**a**) Voltage profiles (first discharge at a current 5 mA g^−1^) of the Cu_*y*_Fe_1-*y*_F_2_ series along with a simple mixture of CuF_2_ and FeF_2_ (**b**) voltage profiles of Cu_0.5_Fe_0.5_F_2_ for the first five cycles (9.2 mA g^−1^), (**c**) cyclic voltammetry (CV) curves for the first (red) and second (black) cycles at a rate of C/40, in comparison to that of FeF_3_ (adapted from ref. 14), (**d**) quasi-equilibrium voltage profile from Cu_0.5_Fe_0.5_F_2_ obtained from galvanostatic intermittent titration technique (GITT) measurements (inset; 150 mA g^−1^ for 3.5 h followed by a 15 h rest). All the measurements were performed at room temperature.

Electrochemical measurements were performed on a series of Cu_*y*_Fe_1-*y*_F_2_ samples to evaluate their electrochemical properties in the presence of two redox centers (Fig. 2). During galvanostatic discharge, Cu_*y*_Fe_1-*y*_F_2_ exhibits a two-step lithiation process as expected (Fig. 2a), but the voltage profiles are different than those obtained from pure CuF_2_, FeF_2_ or a mixture of the two. In Cu_*y*_Fe_1-*y*_F_2_, the Cu conversion (higher plateau) occurs at similar potentials as CuF_2_, while the Fe conversion (lower plateau) occurs at a much higher potential and does not exhibit the voltage dip typically observed in pure FeF_2_, indicating a more facile Fe conversion.^15^ Even at low Cu concentration (for example, 10%), significantly higher rate capabilities were achieved in Cu_0.1_Fe_0.9_F_2_ at room temperature (Supplementary Fig. 4). Similar to other solid–solution systems^26^,^27^,^28^, the electrochemical properties in the ternary system, Cu_*y*_Fe_1-*y*_F_2_, are significantly affected by the cooperative redox of Cu and Fe sitting on the same lattice.

Electrochemical analysis of Cu_0.5_Fe_0.5_F_2_ over the voltage range of 1.0–4.5 V (Fig. 2b) revealed an initial discharge capacity is ~575 mAh g^−1^, comparable to the theoretical value (549 mAh g^−1^ for two electron transfer), and a charge capacity 543 mAh g^−1^ (~94% of the initial discharge capacity), indicating the reoxidization of both the iron and the copper. The reaction process during the subsequent charge and discharge appear to be different than that during the first discharge, as evidenced by the change from two obvious plateaus (~2.9 and ~2.2 V) to three plateaus (~2.8, 3.4, 3.8 V). On subsequent cycles the voltage profiles become similar, indicating a high cycling reversibility. The redox reactions in the Cu_0.5_Fe_0.5_F_2_ electrodes were also investigated by cyclic voltammetry (CV), as given in Fig. 2c, and compared with FeF_3_ (ref. 14). During charge, the first peak is attributed to Fe^0/2+^ oxidation (at ~2.8 V), while the second, located at ~3.4 V, is likely attributed to the further oxidization into trivalent iron (Fe^3+^). The third peak at higher voltage (~3.8 V) is noticeably absent in the CV from FeF_3_ and must be related to Cu oxidation since there are no other redox centers in this voltage range. There are also three peaks in the 2nd discharge, with the first two associated with Fe^2+/0^ and Fe^3+/2+^ reduction and a 3rd at ~3.4 V assigned to Cu^2+/0^ reduction (with the voltage slightly lower than the theoretical value of 3.5 V). The voltage of Cu^2+/0^ reduction during the 1st discharge is relatively low (only about 2.9 V), which is due to a kinetic effect common in conversion reaction electrodes^15^. In contrast to pure CuF_2_, which showed no reversible redox Cu peaks, the redox peaks in Cu_0.5_Fe_0.5_F_2_ are present over multiple cycles, indicating different electrochemical behaviour in the solid–solution ternary phase (See Supplementary Fig. 5 and Supplementary Note 2 for comparison of Cu redox reactions between CuF_2_ and Cu_0.5_Fe_0.5_F_2_).

Another striking feature observed in the cycling data is the small voltage hysteresis. Even during conventional galvanostatic cycling (Fig. 2b), the measured voltage gaps are only ~0.48 V for Cu^0/2+^, ~0.63 V for Fe^0/2+^ and ~0.43 V for Fe^2+/3+^. Those values are significantly less than that of binary fluorides, such as FeF_2_, which is ~1.6 V (see Supplementary Fig. 6. The voltage hysteresis measured by galvanostatic intermittent titration technique (GITT) is reduced to 148 mV for the Cu^0/2+^ redox and ~200 mV for the Fe redox (Fig. 2d), which is substantially lower than pure FeF_2_ (700 mV)^20^ and comparable to intercalation-type electrodes. This is the lowest reported hysteresis for conversion reaction in any metal fluoride, indicating the potential for achieving high-energy efficiency in ternary fluoride cathodes. In addition, these results also suggest that the hysteresis is not solely determined by the anions, but is also affected by the type of cations present. This is further verified by the different thermodynamic and kinetic behaviours between Cu_0.5_Fe_0.5_F_2_ and pure FeF_2_, CuF_2_ (Supplementary Figs 4 and 7 and Supplementary Note 3).

### Reversibility of redox reactions in Cu_*y*_Fe_1-*y*_F_2_

**Figure 3:**
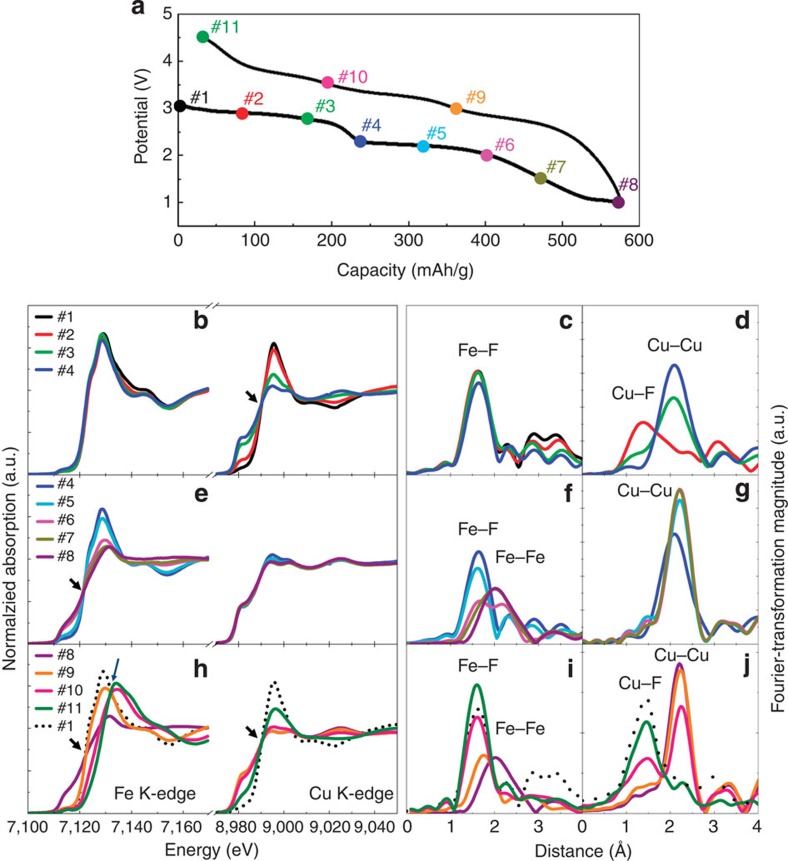
Reversible redox reactions of Cu and Fe in Cu_0.5_Fe_0.5_F_2_. (**a**) Typical voltage profile of Cu_0.5_Fe_0.5_F_2_ for the first cycle with labels of various (de)lithiated states (#1-#11) for samples used in XAS measurements, (**b**–**j**) near-edge XAS spectra (XANES) and Fourier transformation (FT) of extended fine structure (EXAFS) for both Fe and Cu edges, with (**b**,**c**,**d**) for the first lithiation stage (#1-#4), (**e**,**f**,**g**) for the second lithiation stage (#4-#8) and (**h**–**j**) for the delithiation process (#8-#11) compared with the pristine material (#1). Isosbestic points in the XANES spectra (indicating two-phase behaviours) are labelled by arrows.

Elemental specific XAS measurements were performed on Cu_0.5_Fe_0.5_F_2_ to gain insight into the Cu and Fe redox reactions and local structural reorganization, and to correlate these results with the electrochemical behaviour. Figure 3 shows the results from XAS near-edge structure (XANES) and extended fine structure (EXAFS) measurements of Fe and Cu K-edges during the 1st cycle. On discharge, the XANES spectra clearly indicate the conversion of Cu occurs first (#1→#4), followed by that of Fe (#4→#8) at lower voltages (Fig. 3b,e). The XANES spectra from the Cu K- (Fig. 3b) and Fe K-edges (Fig. 3e) reveal an isosbestic point (as labelled by an arrow), indicating a two-phase transition behaviour of the conversion reactions. Simultaneous dissociation of Cu–F/Fe-F bonds and the formation of metallic Cu-Cu/Fe-Fe bonds at each plateau were also confirmed by the Fourier transformation (FT) of the EXAFS (Fig. 3c,d,f,g). XRD measurements (Supplementary Fig. 8) also show decomposition of the initial solid–solution phase and formation of metallic Cu^0^ after the high-voltage plateau, while there is no visible diffraction peak from FeF_2_, indicative of the highly disordered nature of the FeF_2_ after Cu conversion. The intermediate FeF_2_ is then reduced to metallic Fe^0^ at lower voltages, (Fig. 3e,f).

The charge process is quite different from the discharge as shown in Fig. 3h–j. At the initial stage of charge (#8→#9), the Fe oxidation state increases from 0 to 2+ (Fig. 3h). On further delithiation (#9→#11), the oxidation state of Fe continues to increase (indicated by edge shift to higher energies), along with the formation of a 2nd isosbestic point indicating the further oxidation of Fe^2+^ to a higher valence state, but only partially (as verified by XANES of Fe K-edge; Supplementary Fig. 9)^29^. This is in agreement with the CV data, which shows a redox peak at ~3.4 V (Fig. 2c). The strong Fe–F peak, with bond distance similar to that of FeF_6_ octahedra in a rutile phase, is evident in the final product (Fig. 3i), suggesting the reconversion back to a rutile-like framework.

**Figure 4:**
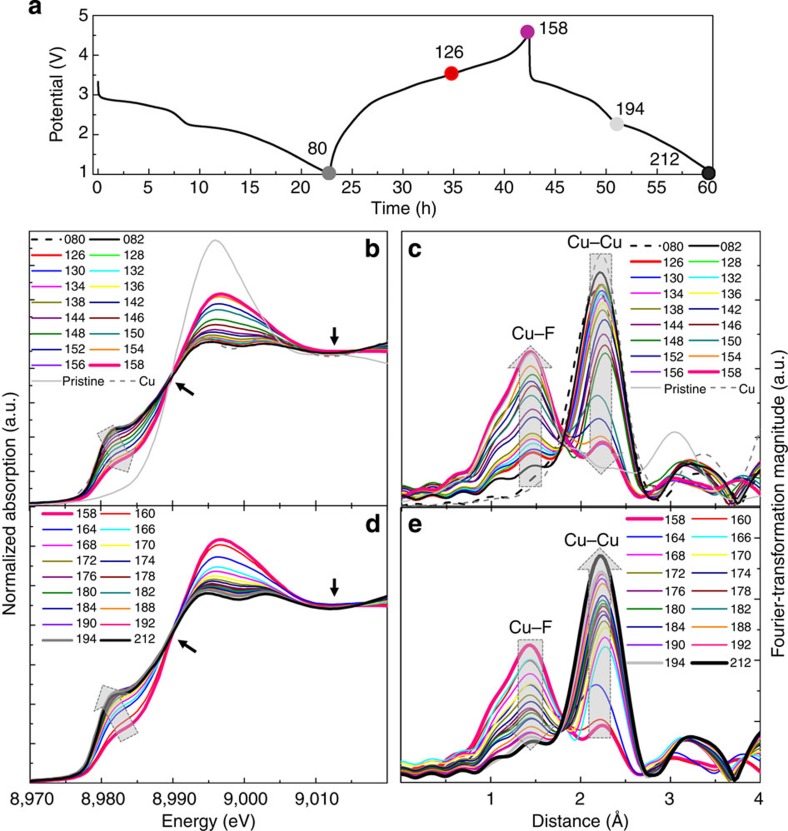
*In situ* XAS of reversible Cu oxidation/reduction in Cu_0.5_Fe_0.5_F_2_. (**a**) Voltage profile for the 1st cycle and 2nd discharge, (**b**,**c**) XANES and FT EXAFS for Cu K-edge during 1st charge (Cu oxidation), (**d**,**e**) XANES and FT of EXAFS for the Cu K-edge during the 2nd discharge (Cu reduction). Isosbestic points in the XANES spectra are labelled by arrows.

In the high-voltage region (above 3.5 V; #10→#11), the shift of the Cu K-edge to higher energies provides direct experimental evidence for oxidization of Cu^0^ back to a high-valence state (Fig. 3h). In addition, the reformation of the Cu–F bonds is evident from the FT EXAFS data (Fig. 3j), showing a strong Cu–F peak with exactly the same position and shape as in the pristine material. Due to the over oxidation of Fe to Fe^3+^ during which extra LiF is consumed, Cu^0^ cannot be fully oxidized into Cu^2+^. But it should be noted that, Fe is only partially oxidized into Fe^3+^ (as verified by XANES of Fe K-edge in Supplementary Fig. 9), allowing much of the Cu to be converted to Cu^2+^, while the rest remains as Cu^0^ (Figs 3j and 4c). These results provide direct verification of a reversible Cu redox (Cu^0/2+^) in Cu_0.5_Fe_0.5_F_2_ (as observed in Fig. 2). This behaviour is different than what is observed in pure CuF_2_, as indicated by the valence state and local coordination of Cu after one complete discharge/charge cycle (Supplementary Fig. 10 and Supplementary Note 4). Although Cu^2+^ is fully reduced to metallic Cu^0^ during the 1st discharge, Cu is only partially oxidized (to a soluble Cu^+^) in pure CuF_2_ during the first charge (delithiation)^23^. The extent of Cu oxidation on charge is significantly higher in Cu_0.5_Fe_0.5_F_2_ (even after four cycles) as evidenced in the Cu K-edge XANES. While the local coordination of Cu in the reconverted CuF_2_ forms a doublet and is distinctly different from that in the pristine CuF_2_, or reconverted Cu_*y*_Fe_1-*y*_F_*y*_, in which only a single Cu–F peak was observed. The Cu valence state and coordination in the reconverted Cu_0.5_Fe_0.5_F_2_ is also different than other Cu^+^ compounds, such as CuCl, but similar to that of “0.5Cu^0^+0.5Cu_0.5_Fe_0.5_F_2_” (Supplementary Fig. 11).

Due to the disordered nature of phases formed during conversion and reconversion in Cu_*y*_Fe_1-*y*_F_*x*_, their structures were not identified from XRD measurements (Supplementary Fig. 8), but well resolved locally by electron diffraction and STEM–EELS (Supplementary Fig. 12 and Supplementary Note 5). The most salient feature of these results is that most of the Cu and Fe are atomically mixed both in the pristine and reconverted states, although some larger (presumably inactive) Cu particles were observed in the EELS maps (Supplementary Fig. 12a–e). The near-edge features of the Cu L-edge, such as the Cu L_3_ peak at ~933 eV, clearly shows that Cu in the reconverted phase is nearly identical to that in the pristine material (Supplementary Fig. 12f); nevertheless the Cu K-edge spectra in the discharged samples (at 2.4 and 1.5 V) show broad plateaus characteristic of metallic Cu (additional details in Supplementary Note 5). These results are consistent with observations in the Cu K-edge XANES and EXAFS measurements, indicating the reconversion of Cu back to a state close to Cu^2+^ (bonded with F). Although no peaks associated with the rutile-like structure were identified by XRD (Supplementary Fig. 8), the electron diffraction pattern, recorded from localized regions of the reconverted Cu_*y*_Fe_1-*y*_F_*x*_ (Supplementary Fig. 12g), shows diffusive rings that are overall similar to those from the pristine sample, indicating the reformation of rutile-like structure in the Cu_*y*_Fe_1-*y*_F_*x*_ electrodes after charge, consistent with the Cu K-edge EXAFS results (Fig. 3j).

### Evolution of Cu in Cu_*y*_Fe_1-*y*_F_2_ during cycling

To track the valence state and local coordination of Cu and better understand Cu redox behaviour in a working electrode, *in situ* XAS measurements (XANES and EXAFS of Cu K-edge) were performed on the Cu_0.5_Fe_0.5_F_2_ electrodes, with hundreds of spectra acquired during the 1st one and half cycles. Since the Cu reduction during the first discharge is well understood, only the results from the first charge and second discharge are presented here (Fig. 4). The results from *in situ* XAS measurements during charge (Fig. 4b,c) reveal a gradual Cu oxidation from Cu^0^ to Cu^2+^ as indicated by the gradual chemical shift to higher energies, and the formation of the Cu–F bonds as indicated by growth of Cu–F peak in the FT of EXAFS (up to an amplitude similar to that of the pristine sample). This process is reversed on discharge (second lithiation) where the Cu K-edge shifts to lower energies and the Cu–F peak in the FT EXAFS data disappears as Cu is reduced back to the metallic state (Fig. 4d,e). This behaviour is distinctly different than what was observed in the CuF_2_ electrode, in which no further reduction was found during the second cycle (Supplementary Fig. 10, and Supplementary Note 4, and also reported in ref. 23). These results provide direct evidence verifying a reversible Cu redox process in the Cu_0.5_Fe_0.5_F_2_ electrode (which does not occur in CuF_2_). In addition, the isosbestic points in the XANES data during the first charge and second discharge suggest the dominant reaction is two phase, involving Cu^0^←Cu^2+^, without going through a Cu^+^ intermediate (such as Cu–F; being consistent with DFT calculations in Supplementary Fig. 13 and Supplementary Note 6). Despite these results, analysis of the internal cell components after cycling indicates that some Cu dissolution (Cu^+^) still occurs in Cu_0.5_Fe_0.5_F_2_ and these parasitic reactions are likely responsible for much of the capacity fade in this system (see Supplementary Fig. 14 and Supplementary Note 7). Various mitigation methods, such as surface coatings to stabilize the electrode at high potentials or barrier layers to prevent crossover, may be useful at limiting the loss of Cu and mitigating the capacity decay^30^,^31^.

Although Cu reoxidization is expected to occur at voltages above 3.55 V during charge (considering the overpotential), the *ex situ* XAS results clearly reveal a slight chemical shift in the Cu K-edge along with the formation of a surprisingly large Cu–F peak in the FT EXAFS in Cu_0.5_Fe_0.5_F_2_ charged to only 3.5 V (with a 10-h hold; Fig. 3h,j). The Cu reoxidization at low potentials is evident in the *in situ* XAS data (Fig. 4), particularly by the formation of a small Cu–F peak in the FT EXAFS (spectrum *#82* in Fig. 4c) at potentials as low as ~1.5 V. This peak occurs almost simultaneously with the Fe reconversion (Fe^0/2+^), and gradually grows into an intense peak at 3.5 V (spectrum *#126)*. These results indicate that Cu reconversion is initiated at low potentials and largely overlaps with Fe oxidation, which may consequently lead to the reformation of the solid–solution phase (Cu_*y*_Fe_1-*y*_F_2_). This newly reformed Cu_*y*_Fe_1-*y*_F_2_ phase has a somewhat disordered structure, but remains a rutile-like framework, similar to the pristine material. This repeatable, cooperative redox behaviour (after the first discharge) may also explain the origin of the reversibility in this system (Fig. 2b,c). The disclosed cooperative redox behaviour in ternary fluorides may also be widely applicable to other systems, such as multication oxides or oxyfluorides^32^,^33^, provided that solid–solution phases can be formed.

## Discussion

**Figure 5:**
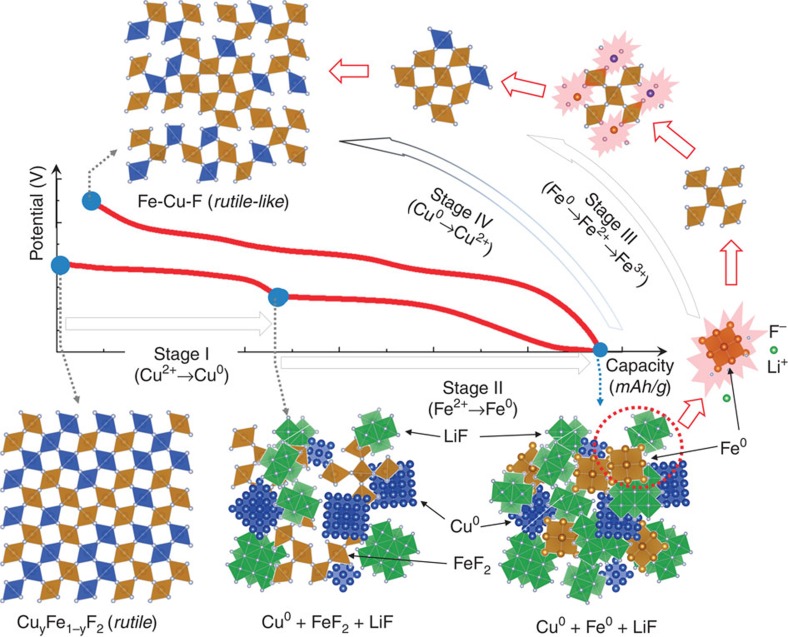
Schematic illustration of the asymmetric reaction pathway in Cu_*y*_Fe_1-*y*_F_2_. Cu and Fe reduction occurs sequentially during discharge (Stages *I* and *II*), while the Fe and Cu oxidation processes (*III* and *IV*) overlap during charge, which enables the reformation of a disordered rutile-like Cu–Fe–F phase.

A summary of the reaction pathway and phase evolution in Cu_*y*_Fe_1-*y*_F_2_ is illustrated in Fig. 5. During the initial discharge, the conversion process occurs in two stages (*I* and *II*), which involve the reduction of Cu and Fe, while the reconversion (*III* and *IV*) is more complicated, and follows a different pathway. The reactions in Stage *III* start with Fe reconversion to FeF_2_, followed by transformation into a rutile-like iron fluoride (with Fe at a valence of Fe^2+/3+^). The reconversion of Cu is initiated at the very beginning of Stage *III*, triggered by the preformed rutile-like framework. Due to the structural similarities, the nucleation and subsequent growth of the Cu-based fluoride phase on the surface of rutile-like iron fluoride likely requires less energy than direct nucleation of CuF_2_, which could reduce the overpotential and enable the reconversion at very low potentials, leading to formation of the Cu–Fe–F-based rutile structure. As the potential is further increased (in Stage *IV*), much of the Cu is reconverted back to the rutile structure, with a small amount of irreversible Cu dissolved into the electrolyte or segregated into larger, isolated particles (see Supplementary Figs 12 and 14 and Supplementary Note 7). So consequently, the converted phase may not be Cu_0.5_Fe_0.5_F_2_, but a Cu-deficient phase, such as Cu_0.35_Fe_0.65_F_2_ or other compositions, as being predicated by DFT calculations (Fig. 1d).

The revealed reaction pathway and correlated local structural reorganization may help to understand the small overpotential and strikingly low voltage hysteresis in Cu_*y*_Fe_1-*y*_F_*x*_ (Fig. 2). First the formation of nanosized FeF_2_ intermediates, surrounded by metallic Cu^0^ from Cu conversion (Supplementary Fig. 12a–e), may accelerate the Fe conversion due to the increased ionic conductivity (resulting from the large LiF/FeF_2_ interface) and the enhanced electronic transport (in the presence of metallic Cu^0^)^15^,^16^. The increase in defects and structural disorder, along with the size reduction of the FeF_2_ after Cu conversion is likely responsible for the higher discharge potential during the initial Fe conversion. Similar observations of elevated conversion potential were also reported in amorphous RuO_2_ (compared with crystalline phase)^34^. The low voltage hysteresis associated with the Cu redox (Fig. 2d) is most likely due to the low nucleation barrier for Cu–F formation on/within the existing Fe–F framework. In addition, the structural disorder of the reformed Cu–Fe–F framework, and the intrinsically high mobility of Cu ions may also play a role.

In conclusion, novel ternary metal fluorides M^1^_*y*_M^2^_1-*y*_F_*x*_ (M^1^, M^2^=transition metal) were prepared by a mechanochemical process to form a variety of solid solutions, which exhibit interesting electrochemical properties. The initial conversion reaction (lithiation) in Cu_*y*_Fe_1-*y*_F_2_ proceeds via a two-stage process, the reduction of Cu to metallic Cu^0^ and concomitant formation of disordered FeF_2_, followed by Fe^2+/0^ reduction. The reformation of the fluoride takes a different path, during which Fe^0^ is partially oxidized up to Fe^3+^, leading to the formation of a rutile framework, which promotes the reconversion of Cu to form a disordered rutile-like Cu–Fe–F final phase (overall similar to the pristine material). However, the formation of some trivalent iron limits the full reconversion of Cu^0^ back to Cu^2+^. Although cation dissolution remains a challenge for the long-term cyclability, the Cu-based ternary fluorides exhibit two truly unique electrochemical properties—a reversible Cu^2+/0^ reaction and remarkably low hysteresis (<150 mV), which, along with intrinsically high voltage and capacity, makes them appealing for use in next-generation rechargeable batteries.

## Methods

### Synthesis of M^1^_*y*_M^2^_1-*y*_F_2_ solid–solution

As-purchased CuF_2_ (Aldrich, 98%), FeF_2_ (Aldrich, 98%), NiF_2_ (Aldrich, 98%) and CoF_2_ (Aldrich, 98%) were used as starting materials without any further purification. A stoichiometric mixture of two MF_2_ compounds was introduced into a stainless steel reactor inside an Ar-filled glove box. The reactor was sealed to prevent air contamination and transferred to planetary ball-mill (Fritsch, Pulverisette 6). The mixed powder was ball-milled at 300 r.p.m. for 12 h. After the milling, the container was opened inside the Ar glove box to collect the final product for characterization.

### DFT calculations

All DFT calculations were performed with the spin-polarized generalized gradient approximation (GGA) within the Perdew–Burke–Ernzerhof (PBE) functional^35^. A plane-wave basis set and the projector-augmented wave method were used, which were implemented in the Vienna *ab initio* simulation package (VASP)^36^. The Hubbard parameters (GGA+U) were used to correct the incomplete cancelation of the self-interaction of the GGA^37^. An effective *U*-value of 5.3 eV for Fe ion and 4.0 eV for Cu ion were used^38^,^39^. A plane-wave basis set with a kinetic energy cutoff of 500 eV and 6 × 4 × 4 Monkhorst-Pack k-point meshes were used to ensure that the total energies converged to less than 5 meV per formula unit. To investigate the phase stabilities of Cu_*y*_Fe_1-*y*_F_2_ (0≤*y*≤1), we calculated all possible Cu/Fe configurations within triple-sized supercells expanded along one of the axes. We considered 135 configurations within the distorted rutile structure and 78 configurations within the tetragonal rutile structure. All symmetrically distinct configurations were generated with a Cluster-Assisted Statistical Mechanics program^40^. Two-hundred and thirteen configurations of different Cu contents were used in calculating the DFT formation energies (as shown in Fig. 1d). The dashed line shows the convex hull of Cu_*y*_Fe_1-*y*_F_2_, when CuF_2_ and FeF_2_ are considered as the end members.

### Characterization of as-synthesized materials

Crystal structures were determined by synchrotron XRD at beam line X14A at the National Synchrotron Light Source (NSLS; *λ*=0.7787 Å). The lattice parameters of the synthesized samples were calculated by Rietveld refinement using the Fullprof software^41^. *In situ* high temperature XRD measurements (up to 250 °C) were also carried out to examine the phase stability. The Cu_0.5_Fe_0.5_F_2_ powder was sealed in a quartz tube in the Ar-filled glove box and resistively heated during XRD measurements. High-resolution (S)TEM images, electron diffraction patterns and EELS mapping were collected from a JEOL TEM machine (JEM 2100F) and a dedicated STEM (Hitachi, HD2700) equipped with an EELS detector (Gatan, Enfina).

### Electrochemical tests

The cycling performance of Cu_*y*_Fe_1-*y*_F_2_ was measured using the conventional composite electrode composed of active materials (72 wt.%), carbon black (18 wt.%) and polyvinylidene fluoride binder (10 wt.%), which were homogeneously mixed together in *N*-methyl-2-pyrrolidone (solvent). The mixed slurry was cast onto an Al foil and dried overnight. All test electrodes were prepared inside the Ar-filled glove box to prevent water absorption. The test electrodes were assembled into CR-2025/2032 type coin cells with Li metal counter electrodes, glass fibre separator (Whatman, GF/D) or a polymer membrane separator (Celgard, 2320) and 1 M LiPF_6_ electrolyte dissolved in 1:1 (by volume) mixture of ethylene carbonate and dimethylcarbonate (DMC). The test cell was cycled using a battery cycler (Arbin Instrument, BT-2400) in constant current mode to collect the electrochemical data. CV measurements were performed using a Solatron 1286 Electrochemical Interface. Galvanostatic intermittent titration technique was performed by applying an intermittent current for 3.5 h followed by a 15 h rest. The pristine cells were cycled between 1.0 and 4.5 V at a current of 150 mA (equivalent to a rate of C/20 at constant current).

### *Ex situ* XRD/XAS/TEM/SEM studies

Cu_0.5_Fe_0.5_F_2_ samples at different (dis)charge states were prepared by controlling the cutoff voltage or the cutoff time during the electrochemical reaction. The test cells after cycling were disassembled using the coin cell disassembler. The cycled electrodes were thoroughly rinsed with DMC and then carefully collected inside the Ar-filled glove. For XRD and XAS measurement, the collected electrodes were sealed inside Kapton tape to minimize air exposure during the measurement.

*In situ* and *ex situ* XAS measurements (Cu K-edge and Fe K-edge) were performed at beam line X18A at the NSLS. The measurements were performed in transmission mode using a Si (111) double–crystal mononchroator. Energy calibration for the absorption edge was made using Fe and Cu foils as a reference (Fe K-edge: 7112 Cu K-edge: 8979). A series of reference spectra (Fe K-edge and Cu K-edge) were recorded from Fe and Cu containing materials, including, FeF_2_, FeF_3_, FeO, Fe_2_O_3_, CuF_2_, CuCl, CuCl_2_, CuO, Cu_2_O. The XAS spectra were analysed using Athena^42^.

TEM samples were loaded onto a TEM holder inside the glove box and then transferred quickly to the TEM to minimize air exposure. The Li metal anode after one cycle was also collected, rinsed with DMC and then attached to carbon tape for SEM-EDS analysis inside the glove box. The SEM holder was sealed and then transferred quickly to the SEM to minimize air exposure.

## Author contributions

F.W. and S.-W.K. conceived and designed the experiments. F.W., S.-W.K., L.W., D.S., J.V., J.W. and J.G. conducted the experiments. D.-H.S. and K.K. performed DFT calculation. F.W. and S.-W.K. made the data analysis and wrote the paper. J.G. assisted with data analysis and writing the paper. All authors were involved in revising the paper.

## Additional information

**How to cite this article:** Wang, F. *et al*. Ternary metal fluorides as high-energy cathodes with low cycling hysteresis. *Nat. Commun.* 6:6668 doi: 10.1038/ncomms7668 (2015).

## Supplementary Material

Supplementary Information Supplementary Figures 1-14, Supplementary Notes 1-7 and Supplementary References
