# Supplementary material for: Ternary metal fluorides as high-energy cathodes with low cycling hysteresis
Source: Nat Commun. 2015 Mar 26;6:6668. doi: 10.1038/ncomms7668 (PMC4389236; doi:10.1038/ncomms7668)
Supplement: Supplementary Information — Supplementary Figures 1-14, Supplementary Notes 1-7 and Supplementary References [file ncomms7668-s1.pdf]

Supplementary Information

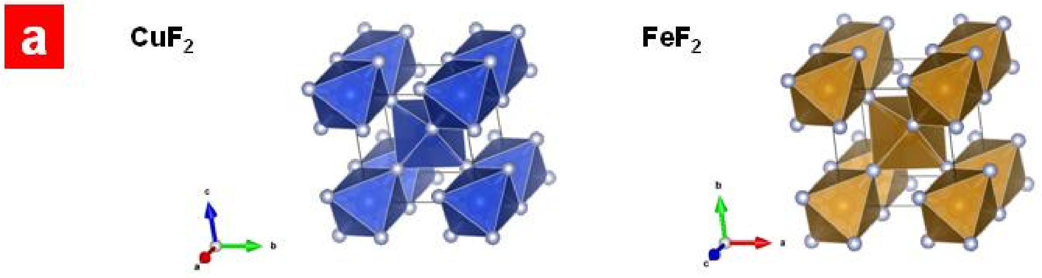

	Space group	a (Å)	b (Å)	c (Å)	β (°)	V (Å³)
CuF_2 (PDF#42-1244)	$P2_1/n$	3.297	4.562	4.616	83.29	68.95
FeF_2 (PDF#45-1062)	$P4_2/mnm$	4.703	4.703	3.306	90	73.12

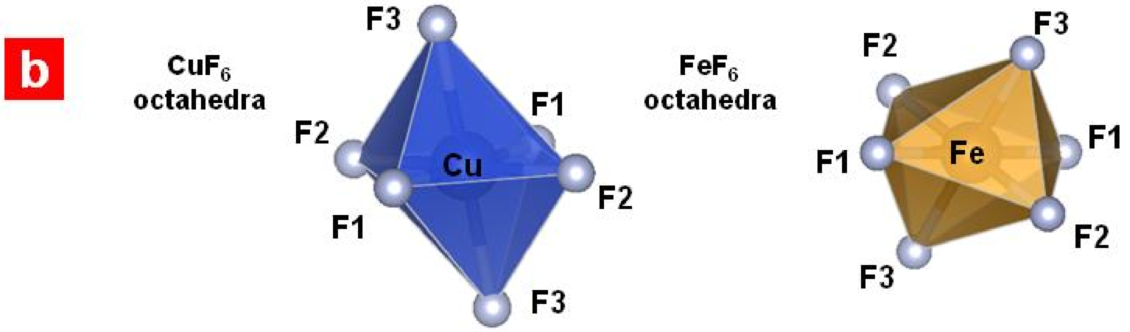

	M-F1 (Å)	M-F2 (Å)	M-F3 (Å)	Average M-F (Å)	Distortion index	Quadratic elongation
Cu-F bond	1.92544	1.92955	2.26445	2.0398	0.07342	1.0313
Fe-F bond	2.01697	2.11089	2.11089	2.0796	0.02007	1.0186

Supplementary Figure 1. Structural similarity between CuF_2 and FeF_2. (a) Crystal structure and lattice parameters and (b) corresponding MF_6 octahedron structure and geometry of CuF_2 and FeF_2 (Cu: blue, Fe: yellow, F: grey).

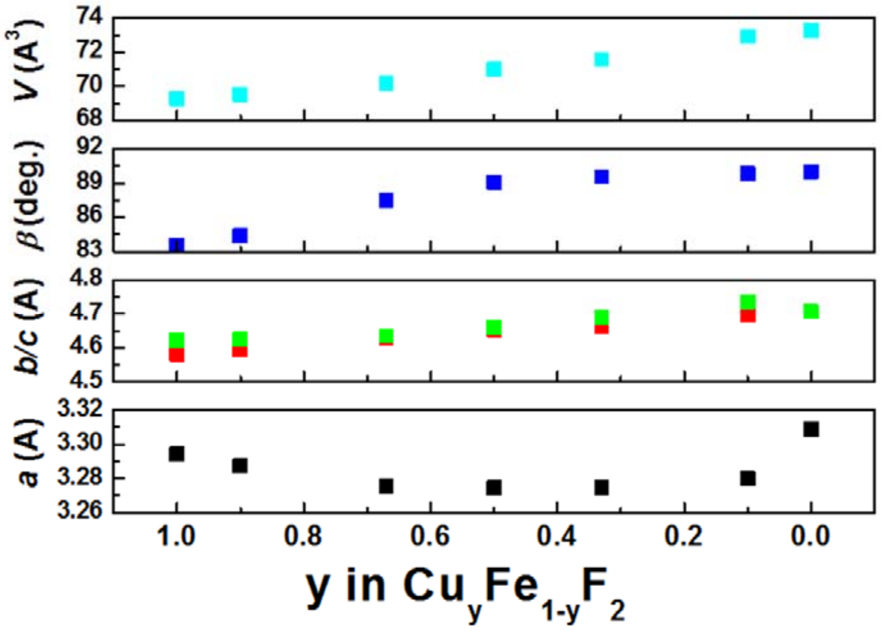

Supplementary Figure 2. Lattice parameters of $Cu_yFe_{1-y}F_2$ series based on CuF_2-like distorted rutile model ($P2_1/n$) except pure FeF_2 (y = 0) ($P4_2/mnm$). *a*-axis in $P2_1/n$ corresponds to c-axis in $P4_2/mnm$ and *b*/*c*-axis in $P2_1/n$ corresponds to *a*/*b*-axis in $P4_2/mnm$. *See also* ***Supplementary note 1***.

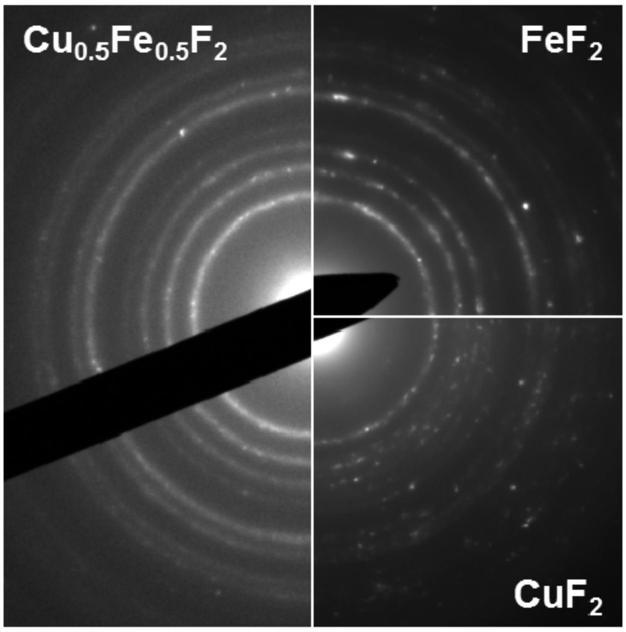

Supplementary Figure 3. Electron diffraction patterns for comparison of the structure between ball-milled $Cu_{0.5}Fe_{0.5}F_2$ (left), FeF_2 (right, upper), and CuF_2 (right, lower). Ring pattern of each sample indicates the formation of nanoparticles. Diffraction pattern of $Cu_{0.5}Fe_{0.5}F_2$ resembles that of rutile FeF_2 without additional diffraction spots. This is consistent with XRD measurements in the main text (Figure 1a). Monoclinic CuF_2 has more complicated diffraction pattern due to less symmetric, monoclinic structure to tetragonal FeF_2.

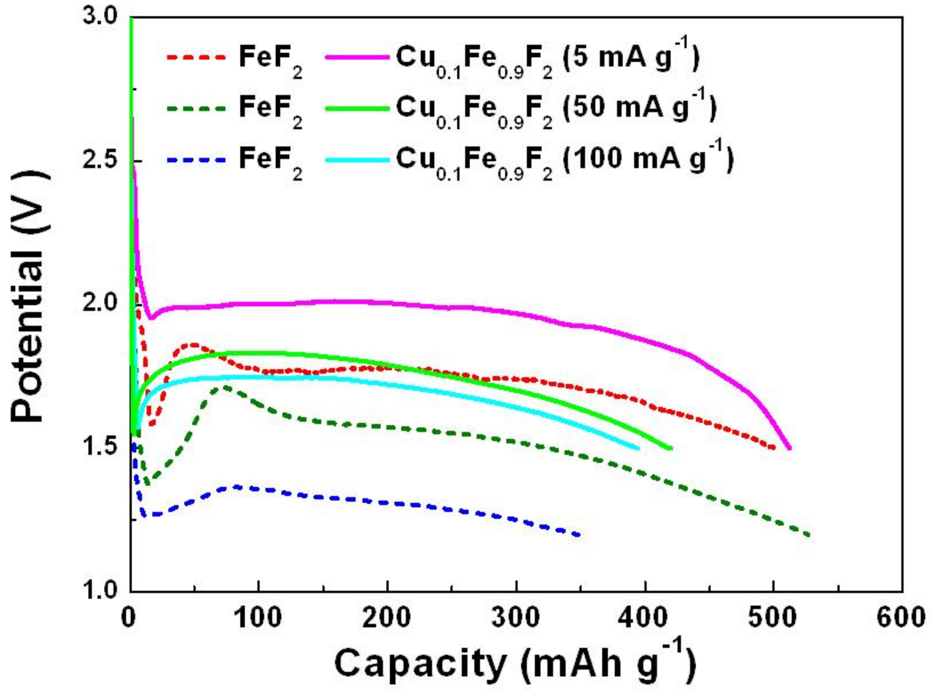

Supplementary Figure 4. Improved reaction kinetics of $Cu_{0.1}Fe_{0.9}F_2$, shown by the much reduced overpotential and voltage dips (in the very beginning of conversion) during gavanostatic lithiation, compared to that of pure FeF_2, at current rates of 5, 50, and 100 mA g^{-1} (cut-off voltage of 1.5 V and 1.2 V for low and high rates). All the measurements were performed at room temperature.

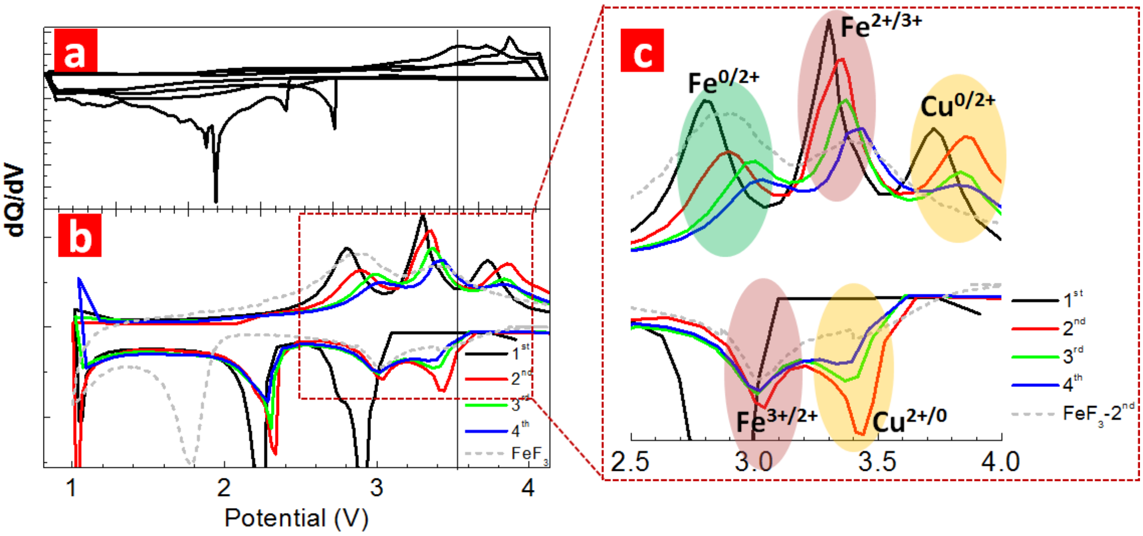

Supplementary Figure 5. Comparison of Cu redox behavior in $Cu_{0.5}Fe_{0.5}F_2$ and pure CuF_2. The derivative of the capacity (dQ/dV) *versus* V, shows the presence of oxidation peaks in CuF_2 only during the 1st charge (a), while oxidation peaks in $Cu_{0.5}Fe_{0.5}F_2$ are present in multiple cycles (b). An expanded view of the reduction/oxidation peaks from $Cu_{0.5}Fe_{0.5}F_2$ and FeF_3 is shown in (c). The dQ/dV curve for the 2nd cycle of FeF_3 (grey) was also added for comparison. *See also* ***Supplementary note 2***.

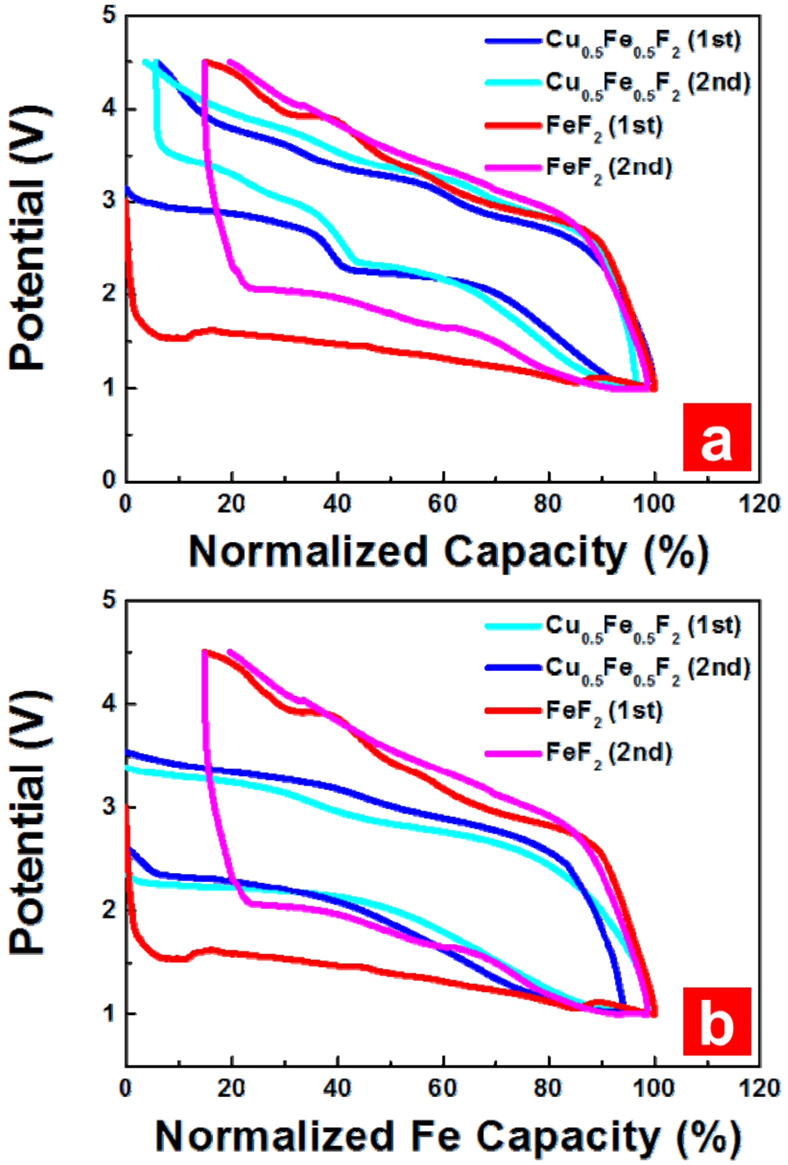

Supplementary Figure 6. Much reduced voltage hysteresis in $Cu_{0.5}Fe_{0.5}F_2$ compared to FeF_2, shown by charge-discharge profiles for the first two cycles. (a) The normalized capacity for the full electrode and (b) the normalized capacity attributed to the conversion reaction of Fe.

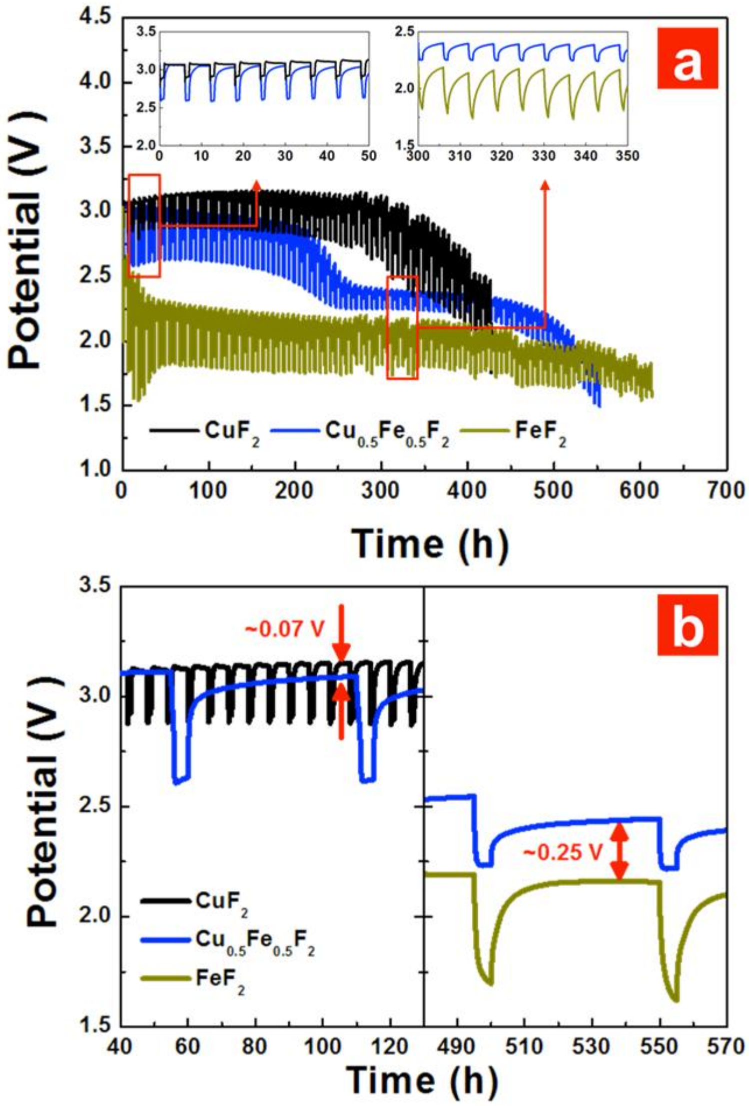

Supplementary Figure 7. (a) GITT profiles of CuF_2, $Cu_{0.5}Fe_{0.5}F_2$ and FeF_2 at identical relaxation time (5 h) and current rate of 5 mA g^{-1} (inset: magnified profiles from two conversion stages), and (b) comparison of GITT profiles between CuF_2 (after 5 h relaxation), $Cu_{0.5}Fe_{0.5}F_2$ and FeF_2 (after 50 h relaxation). All the measurements were performed at room temperature. *See also* ***Supplementary note 3***.

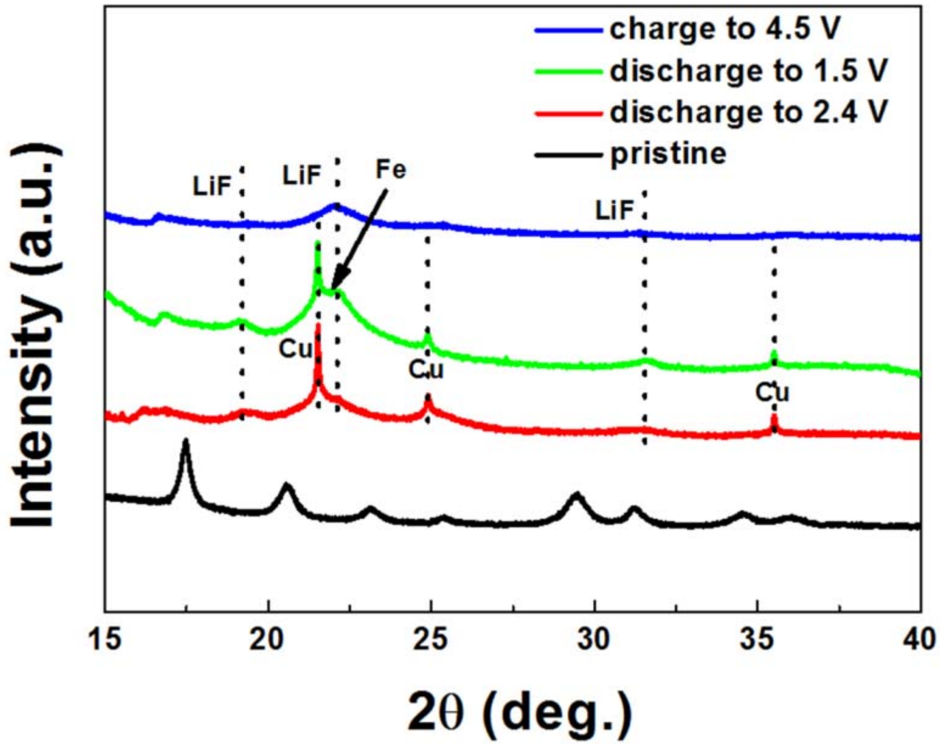

Supplementary Figure 8. Structural analysis of $Cu_{0.5}Fe_{0.5}F_2$ at different (de)lithiated states using synchrotron XRD patterns. The initial rutile structure (with strong peaks) was completely destroyed after discharge to 2.4 V, leading to the formation of metallic Cu^0 (sharp) and LiF (broad). With further lithiation (to 1.5 V), a broad diffraction peak at around 22º, associated with metallic Fe is identified. After charge (4.5 V), peaks associated with Cu^0 and Fe^0 phases mostly disappear, but no other crystalline phase can be detected, due to the disordered nature of the reconverted phase.

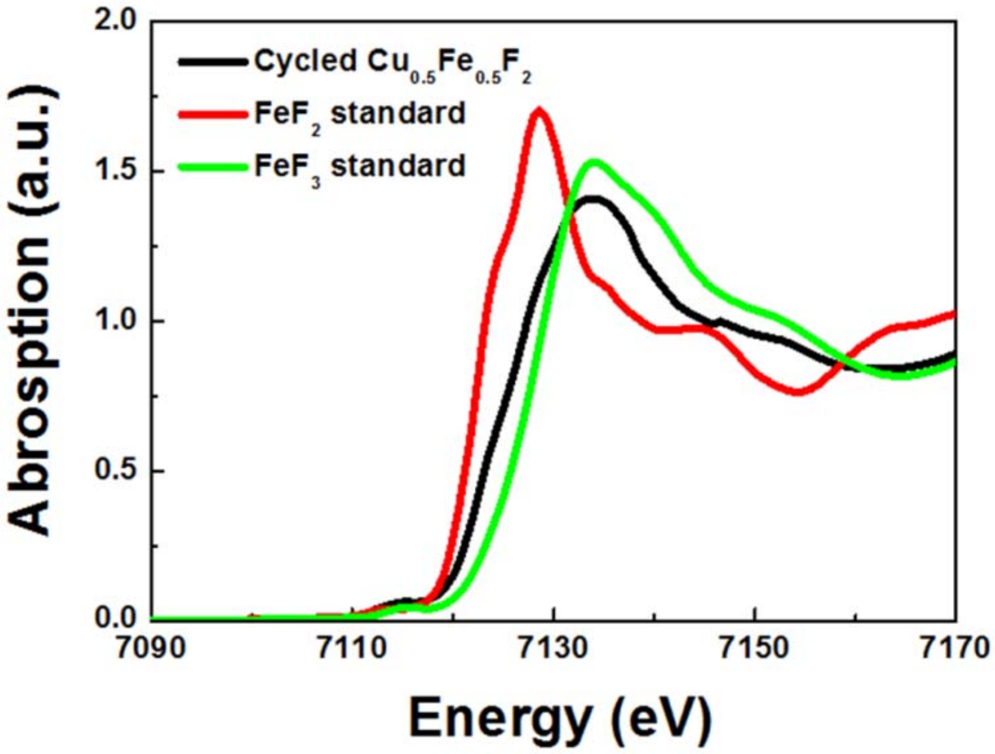

Supplementary Figure 9. Identification of Fe valence state in $Cu_{0.5}Fe_{0.5}F_2$ after one cycle, *vi a* Fe K edge XANES in comparison to that of FeF_2 and FeF_3 standards. Three samples share an i sobestic point, indicating the coexistence of Fe^{2+} and Fe^{3+} in the reconverted phase ($Cu_yFe_{1-y}F_x$) after one cycle.

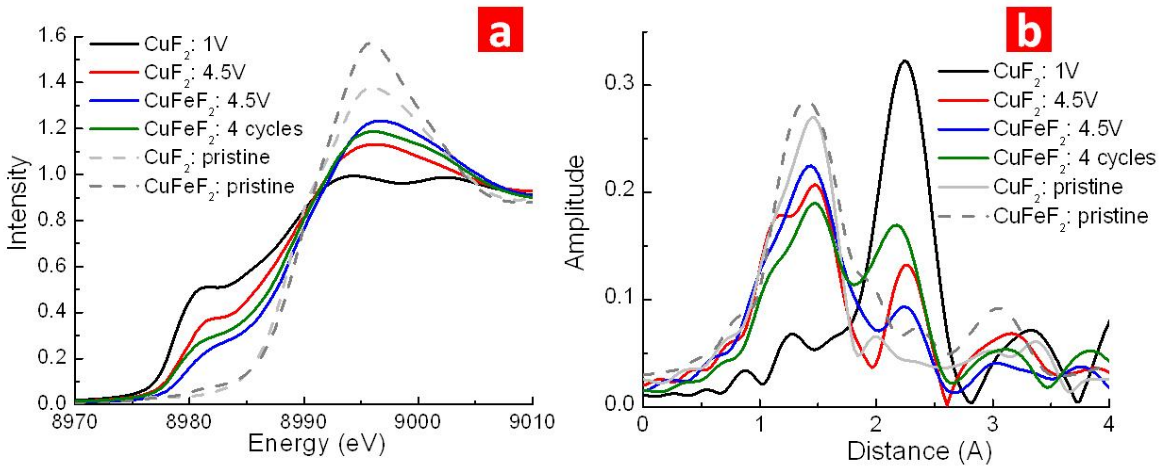

Supplementary Figure 10. **Ex-situ XAS for identifying different reactions between CuF_2 and $Cu_{0.5}Fe_{0.5}F_2$.** a) XANES and b) FT of the EXAFS of Cu K-edge for CuF_2 and $Cu_{0.5}Fe_{0.5}F_2$ at pristine and different lithiated/delithiated states (as labeled). *See also* ***Supplementary note 4***.

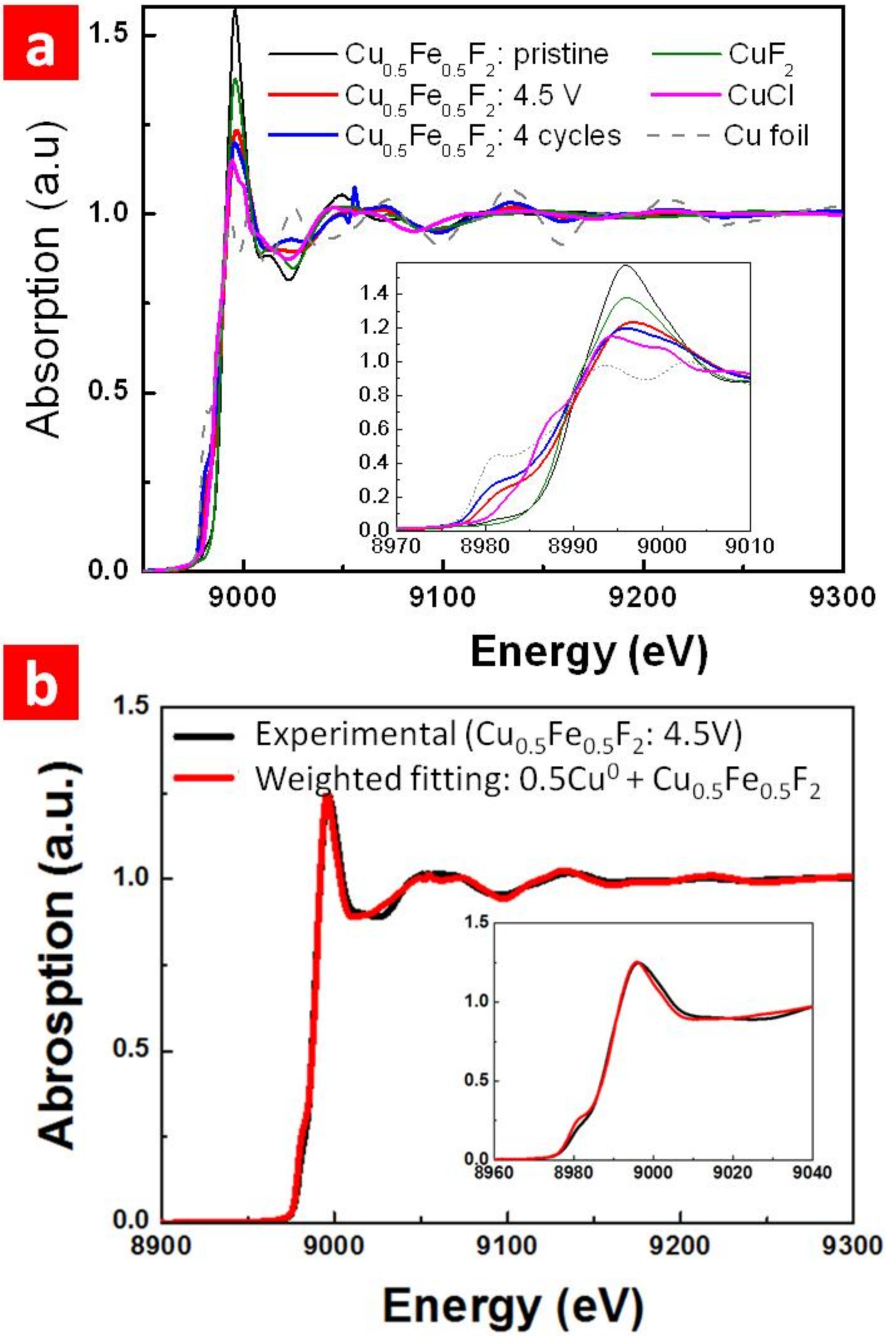

Supplementary Figure 11. Identification of Cu valence state in the reconverted $Cu_{0.5}Fe_{0.5}F_2$ electrodes, *via* comparison of the Cu K-edge XAS spectra of reconverted $Cu_{0.5}Fe_{0.5}F_2$ electrodes to the references (a), and to the fitting results of Cu K-edge of reconverted $Cu_{0.5}Fe_{0.5}F_2$ weighted using 1:1 mixture of Cu^0 and CuF_2 (b).

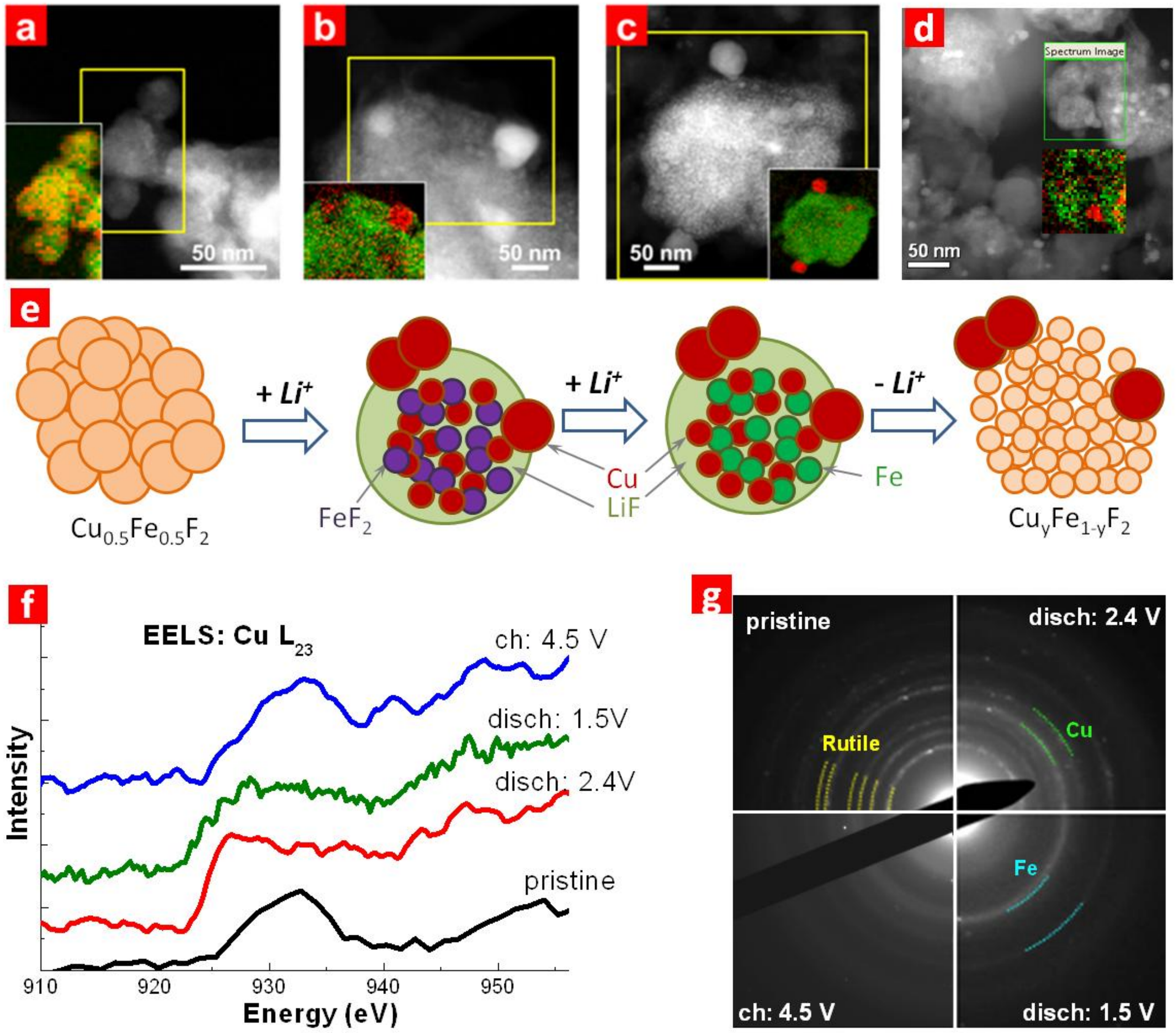

Supplementary Figure 12. Chemical and structural evolution with lithiation and delithiation: (a-d) annular dark field images and (inset) corresponding EELS mapping (red: Cu, green: Fe) results on $Cu_{0.5}Fe_{0.5}F_2$ at pristine state (as-synthesized; *a*), 2.4 V-discharged state (after Cu conversion; *b*), 1.0 V-discharged (after Fe conversion; *c*), and fully charged states (4.5V; *d*), e) the schematic illustration on the morphological evolution of $Cu_yFe_{1-y}F_2$, f, g) Cu K-edge EELS spectra, electron diffraction patterns of $Cu_yFe_{1-y}F_2$ at different lithiated/delithiated states (as labeled). *See also* ***Supplementary note 5***.

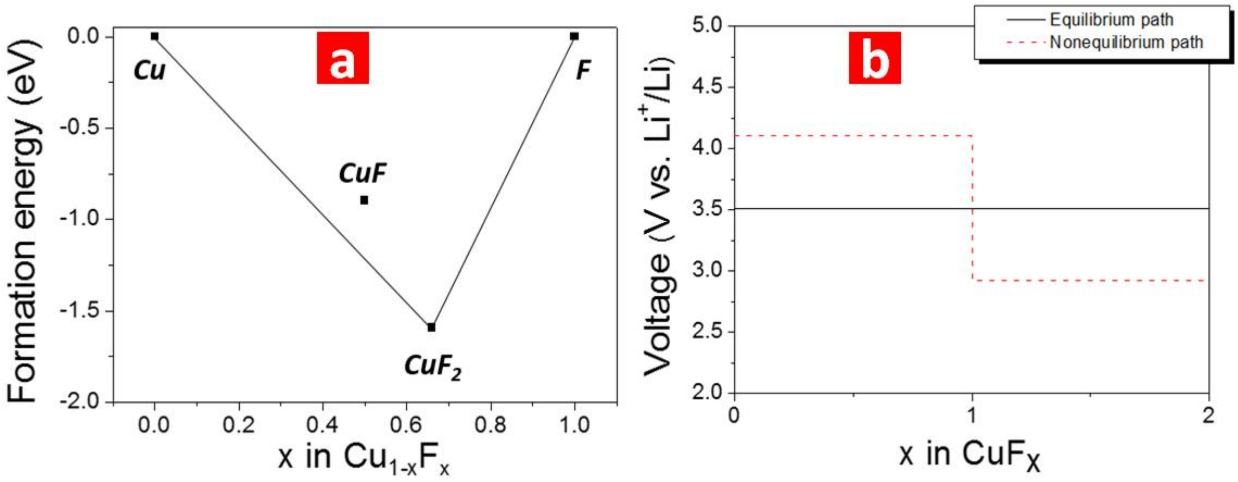

Supplementary Figure 13. (a) formation energies of $Cu_{1-x}F_x$ as a function of its F content ($0 \leq x \leq 1$), (b) the calculated voltages of reconversion reactions of CuF_x ($0 \leq x \leq 2$). *See also* ***Supplementary note 6***.

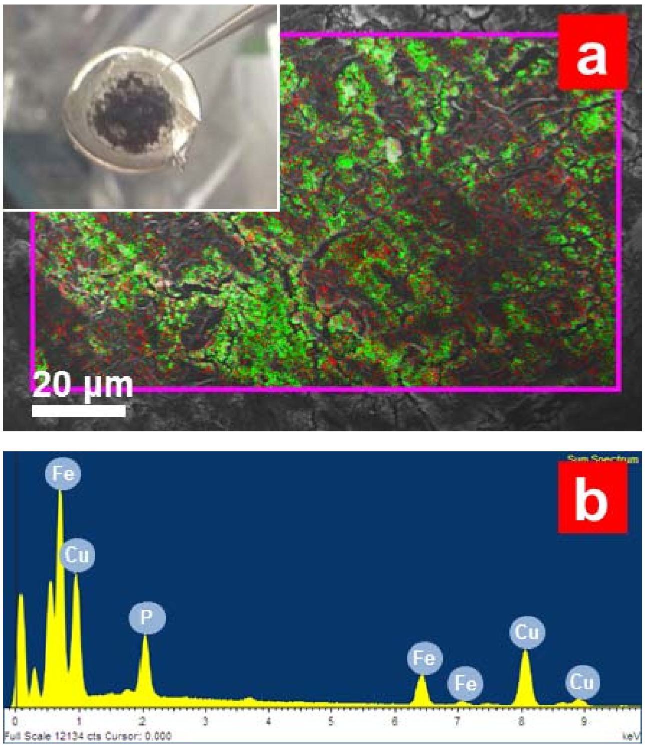

Supplementary Figure 14. (a) SEM-EDS mapping analysis of Li metal anode after one cycle (green: Cu, red: Fe) using $Cu_{0.5}Fe_{0.5}F_2$ as working electrode (inset: photograph image of cycled Li metal anode) and (b) corresponding energy dispersive spectra. *See also* ***Supplementary note 7***.

Supplementary Note 1: Lattice parameters change in $Cu_yFe_{1-y}F_2$ solid solution

Lattice parameters of $Cu_yFe_{1-y}F_2$ were evaluated by profile matching based on the CuF_2-based monoclinic model (space group: $P2_1/n$) except pure FeF2 (space group: $P4_2/mnm$) as shown in Supplementary **Figure 2**. The *β* angle gradually increases with Fe content until it reaches 90°, indicating the structural change from the distorted monoclinic to symmetric tetragonal rutile at higher Fe concentrations. Unit cell volume and *b*/*c* lattice parameters (corresponding to *a*/*b* parameters in the FeF_2-based tetragonal rutile) increase continuously at higher Fe content due to differences in ionic size of Fe^{2+} (92 pm) and Cu^{2+} (87 pm). On the contrary, *a* lattice parameter (corresponding to *c* lattice parameter in the tetragonal rutile) decreases up to y = 0.5 and then becomes larger gradually, which may be attributed to the change in the *β* angle, *i.e.*, the inter-axial angle of *ac* plane.

Supplementary Note 2: Cu redox behavior in $Cu_{0.5}Fe_{0.5}F_2$ and pure CuF_2

To further elucidate the differences in the Cu reactions between the pure CuF_2 and $Cu_{0.5}Fe_{0.5}F_2$, electrodes of each type were fabricated in the same way for electrochemical and structural analysis. As shown in Supplementary **Fig. 5** (dQ/dV versus V), the reduction of CuF_2 occurs at ~2.9 V during the first discharge, followed by oxidation peaks between 3.4-3.7V (charge) attributed to the irreversible Cu+ dissolution/replating. No reduction or oxidation peaks were observed within this voltage range on the 2nd cycle, confirming the irreversible nature of this reaction. These results are consistent with the recent report from Grey et al..[S1] In contrast, the oxidation peak in $Cu_{0.5}Fe_{0.5}F_2$ occurs at a slightly higher potential, 3.72V, in the 1st charge (Supplementary **Fig. 5b**). The peak moves to higher voltage and slowly decreases in intensity in the following cycles, similar to the peaks associated with the Fe redox. Despite intensity decay the peak is still present on the 4th cycle and is clearly distinct from the $Fe^{2+/3+}$ peak (separated by ~350 mV). (Supplementary **Fig. 5c**). These results clearly demonstrate that the Cu redox reaction in the $Cu_{0.5}Fe_{0.5}F_2$ sample is considerably different than those occurring in the pure CuF_2, which are primarily attributed to the irreversible dissolution and replating of Cu^+.

Supplementary Note 3: Thermodynamic and kinetic origins of hysteresis in $Cu_{0.5}Fe_{0.5}F_2$

In the galvanostatic experiments (as in **Figure 2**), the measured voltage is not solely determined by the intrinsic chemical potential (or thermodynamics), but strongly influenced by kinetics. To separate the intrinsic and kinetic factors, galvanostatic intermittent titration technique (GITT) measurements were performed on $Cu_{0.5}Fe_{0.5}F_2$, also on CuF_2 and FeF_2 under the same condition for comparison. Some of the results were given in Supplementary **Figure 7**. The voltage difference before and after relaxation denotes the degree of polarization applied upon the lithiation. As shown in Figure 7a, the quasi-equilibrium state was reached quickly in Cu conversion in CuF_2 (*i.e.*, small polarization) but not in Fe conversion in FeF_2 or $Cu_{0.5}Fe_{0.5}F_2$ within the same relaxation time (5 h). This may be explained by the high diffusivity of Cu atoms, which is slightly reduced in $Cu_{0.5}Fe_{0.5}F_2$, likely due to the presence of neighboring Fe, *i.e.*, hindering diffusion of Cu. It is also remarkable that, in spite of the not-saturated voltage, the polarization of Fe conversion is reduced in $Cu_{0.5}Fe_{0.5}F_2$ (by ~0.21 V) compared to FeF_2, demonstrating the improved Fe conversion kinetics in the solid solution.

GITT performed with longer relaxation time (current applied for 5 h with 50 h rest) was carried out on $Cu_{0.5}Fe_{0.5}F_2$ and pure FeF_2 to get the quasi-equilibrium state. The results (Figure 7b) showed different quasi-equilibrium voltages between pure and the solid solution systems. The voltage of Fe conversion in $Cu_{0.5}Fe_{0.5}F_2$ is higher by ~0.25 V than that in FeF_2, although the difference in Cu conversion is small (~0.07 V). This suggests that the two cations in the same lattice influence each other on the intrinsic thermodynamic behavior (in addition to the kinetics). This may be explained by local phase reorganization according to STEM-EELS measurements (see details in Supplementary Information Fig. 13). The much improved reaction kinetics in Fe conversion is due to the formation of nanosized FeF_2 intermediate surrounded by metallic Cu^0 after the Cu conversion that was directly viewed by EELS mapping (Fig. 13). Such nanostructure is believed to accelerate the Fe conversion reaction because of increased ionic conductivity *via* the massive LiF/FeF_2 interface and the enhanced electronic transport attributed to metallic Cu^0.

Supplementary Note 4: *Ex-situ* XAS of CuF_2 (vs. $Cu_{0.5}Fe_{0.5}F_2$)

Ex-situ XAS measurements were performed to determine the Cu valence and coordination in pure CuF_2 at the fully discharged and charged states (1.0 and 4.5 V respectively, with a constant voltage hold for 10 hours). As shown in Supplementary **Fig. 10a**, a large shift to lower energies is observed in the Cu K edge during discharge, indicating the Cu is fully reduced (to Cu^0). However, the Cu K-edge shift during charge is small, similar to that reported by Grey et al (in *Ref. 1*), but distinctly different than the shift in the reconverted $Cu_yFe_{1-y}F_y$, material (blue curve), in terms of the shift amplitude and shape. A significant chemical shift of the Cu K-edge can still be observed in the reconverted $Cu_yFe_{1-y}F_y$ even after 4 cycles (green curve).

The local environment around the Cu (as observed by EXAFS) is also distinctly different in CuF_2 and $Cu_yFe_{1-y}F_y$ during charge and discharge. After the charge step, Cu in $Cu_yFe_{1-y}F_y$ exhibits a well-defined single Cu-F peak, indicative of the reformation of the metal fluoride, while the Cu in CuF_2 exhibits a doublet indicative of a more complex local coordination (Supplementary **Fig. 10b**).

Supplementary Note 5: Microstructural and chemical evolution in $Cu_{0.5}Fe_{0.5}F_2$ with lithiation/delithiation

The electronic/ionic transport and reaction kinetics has a strong dependency on the local structure. STEM-EELS was used to measure the local structure and chemical distribution (at sub-nm scale) in the $Cu_{0.5}Fe_{0.5}F_2$ solid solution, and their evolution during lithiation (Supplementary **Figure 12**). Cu and Fe are homogeneously mixed in the pristine particle, as expected (Figure 12a). In the partially lithiated state after Cu conversion (Supplementary Figure 12b), small nanoparticles in the main matrix are composed of Fe and F (possibly FeF_2 and LiF) along with Cu well-mixed in the agglomerate (as shown by the mapping in Supplementary Figure 12b). In addition, large Cu^0 particles were observed in the agglomerates. It is speculated that the highly mobile Cu atoms were readily diffused to form the large particles during lithiation, while FeF_2 remained stable in the mother particle (*i.e.*, LiF matrix). At a lower voltage (1.0 V), more Li ions were inserted and nanosized FeF_2 was fully converted to Fe^0 (brighter contrast), which is well dispersed in the LiF matrix (Supplementary Figure 12c). The converted Fe^0 particles were mostly less than 5 nm. This result agrees well with our previous work on the two separated CuF_2 and FeF_2 systems[Ref. 15 in the main text] and indicates that the particle size of the conversion reaction product is mainly determined by the intrinsic property of the transition metal element (*e.g.*, mobility and surface energy). In the fully reconverted state (Supplementary Fig. 12d), Cu and Fe become highly mixed again, although some larger (presumably inactive) Cu particles were observed. Supplementary Figure 12e briefly illustrates the morphological evolution and corresponding phase separation during the lithiation.

According to the near-edge feature of the Cu L-edge spectra, such as strong Cu L_3 peak at about 933 eV, which is nearly identical in the reconverted phase and pristine. In contrast, the Cu L_3 in the discharged samples (at 2.4 V and 1.5 V) appears at a lower energy (of about 931 eV) and shows a broad plateau. These results are consistent with Cu K-edge XANES and EXAFS, indicating the reconversion of Cu back to a state close to Cu^{2+} (bonded with F).

Due to the disorder of the reconverted phase, no clear peaks associated with a rutile-like structure are identified by XRD (Supplementary Fig. 8). However, the electron diffraction pattern of the reconverted $Cu_yFe_{1-y}F_x$ (Supplementary Fig. 12g), show diffusive rings that are

similar to those from the pristine samples, indicating the formation of a rutile-like structure after reconversion. Again, this is consistent with re-formation of Cu-F bonds as identified by Cu K-edge EXAFS results (Fig. 3j in the main text and Fig. 10 in the supplementary).

Supplementary Note 6: DFT calculations on possible Cu conversion pathway

DFT calculations were performed on the redox potentials using GGA+U, revealing that the redox of $Cu^{2+/0}$ *via* forming CuF_2 like phase is energetically more favorable than the $Cu^{1+/0}$ redox *via* forming intermediate CuF.

The average voltages of the conversion reaction were calculated with the energies of the involved compounds.[Ref. 2]

The overall conversion reaction of MF_2 can be written as

$$MF_2 + 2Li^+ + 2e^- \rightarrow M + 2LiF \quad (1)$$

For this conversion reaction, the average voltages can be simply determined by using following equation:

$$\langle V \rangle = -[2E(LiF)+E(M)-E(MF_2)-2E(Li)]/2F \quad (2)$$

where $E(LiF)$, $E(MF_2)$, $E(M)$, and $E(Li)$ are the ground state DFT energies of each compound /elemental metal and F is the Faraday constant.[Ref. 3] We combined the GGA and GGA+U energies to predict the redox potentials of these conversion reactions. The energies of metallic systems such as Fe and Cu were calculated with the GGA methodology due to their delocalized electronic states of their *d* orbital, whereas the energies of metal oxide compounds such as FeF_2 and CuF_2 were calculated with the GGA+U due to their localized electronic states. We calibrated the energies using a correction term to use the GGA and GGA+U energies together because of the discrepancy between the GGA and GGA+U energies.[Ref. 4] We used pseudopotentials of Li, F, Fe_pv and Cu. The correction term is -2.733 eV for FeF_2 and -1.156 eV for CuF_2.[Ref. 3, 5] The calculated average voltages of conversion reaction of FeF_2 and CuF_2 compounds are 2.270 and 3.511 V, respectively, which are in good agreement with our experimental results. We also calculated redox potentials of $Fe_{0.5}Cu_{0.5}F_2$ with following conversion reactions:

$$Fe_{0.5}Cu_{0.5}F_2+2Li \rightarrow 0.5Cu+LiF+0.5FeF_2+Li \rightarrow 0.5Cu+0.5Fe+2LiF \quad (3)$$

The redox potential of each step of conversion reaction is 3.472 V and 2.270 V in the sequence of reaction; being also consistent with our measurements.

We also considered CuF compound (F-43m, pdf number: 01-071-3775) as an intermediate compound of conversion reaction during charging. The energy above hull of CuF is around 0.6 eV / f.u. (f.u. = CuF), as shown in Supplementary **Figure 13a**, indicating that this

compound is energetically unstable than the mixture of Cu and CuF_2. When we assume that CuF is kinetically formed with F reinsertion to Cu, the redox potential of this nonequilibrium conversion reaction should be higher than that of equilibrium one. We calculated redox potentials of these reactions with first principles calculations (see also Supplementary **Fig. 13b**) as follows:

$$LiF + Cu \rightarrow Li + CuF \ (4.104\ V) \qquad (4)$$

$$LiF + CuF \rightarrow Li + CuF_2 \ (2.919\ V) \qquad (5)$$

The calculated redox potential of Cu^0/Cu^+ in this system (4.104 V) is much higher than our experimental result (~3.8V). If we consider polarization of conversion reaction, the experimental value is rather similar to calculated redox potential of Cu^0/Cu^{2+} (3.51V). Therefore, we speculated that the reconversion of LiF and Cu nanoparticles take place without CuF formation.

Supplementary Note 7: SEM-EDX investigation of Cu dissolution in $Cu_{0.5}Fe_{0.5}F_2$ during cycling

As shown in Figure 2b in the main text, the specific capacity of $Cu_{0.5}Fe_{0.5}F_2$ decays with cycling, which may be largely due to the Cu dissolution (and consequently the degradation of the Li counter electrode) (inset of Supplementary Figure 14a). Scanning electron microscope-energy dispersive spectroscopy (SEM-EDS) was performed on the Li anode after cycling to determine the chemical species present on the surface. The presence of Cu and Fe identified by elemental analysis indicates the dissolution of Cu and Fe with cycling. These results suggest that the Cu and Fe are oxidized to form soluble cationic species (*e.g.*, metal ions or metal-electrolyte complex ions) in the electrolyte, which migrate to the anode upon charge. The Cu content is much higher than that of Fe content, indicating the vulnerability of Cu (maybe due to formation of Cu^{1+}) to the dissolution compared to that of Fe.

Supplementary References

Ref.1: X Hua, et al., "Comprehensive Study of the CuF2 Conversion Reaction Mechanism in a Lithium-ion Battery, Journal of Physical Chemistry 118 (2014) 15169.

Ref.2: H. Li et al., J. Electrochem. Soc. 151 (2004) A1878 and M. Oh et al., Science 340 (6135) (2013) 964.

Ref.3: T. H. Mason et al., J. Phys. Chem. C **115** (2011) 16681.

Ref.4: A. Jain et al., Phys, Rev, B **84** (2011) 045115.

Ref.5: S. P. Ong et al., Comp. Mater. Sci. **68** (2013) 314.
